# Restless Leg Syndrome in Peripheral Artery Disease: Prevalence among Patients with Claudication and Benefits from Low-Intensity Exercise

**DOI:** 10.3390/jcm8091403

**Published:** 2019-09-06

**Authors:** Nicola Lamberti, Pablo Jesús López-Soto, María Aurora Rodríguez-Borrego, Sofia Straudi, Nino Basaglia, Paolo Zamboni, Roberto Manfredini, Fabio Manfredini

**Affiliations:** 1 Department of Biomedical and Surgical Specialties Sciences, University of Ferrara, 44121 Ferrara, Italy; 2 Department of Nursing, Maimonides Biomedical Research Institute of Cordoba (IMIBIC), University of Cordoba, Reina Sofía University Hospital, 14004 Cordoba, Spain; 3 Unit of Physical and Rehabilitation Medicine, University Hospital of Ferrara, 44121 Ferrara, Italy; 4 Unit of Translational Surgery, University Hospital of Ferrara, 44124 Ferrara, Italy; 5 Department of Medical Sciences, University of Ferrara, 44121 Ferrara, Italy

**Keywords:** peripheral artery disease, restless leg syndrome, exercise therapy, rehabilitation, spectroscopy, near-infrared

## Abstract

Restless leg syndrome (RLS) disrupts sleep, affecting the quality of life of patients with various chronic diseases. We assessed the prevalence of RLS in peripheral artery disease (PAD) patients and the effects of a pain-free exercise program. A total of 286 patients with claudication were enrolled in a home-based low-intensity exercise program prescribed at the hospital. RLS was determined through standardized questions. Hemodynamics, degree of calf deoxygenation, and mobility were assessed using the ankle-brachial-index, a treadmill test assisted by near-infrared spectroscopy and the 6-min walk test, respectively. During hospital visits, persistence of RLS, adherence to exercise, hemodynamics, and mobility were assessed. At the enrollment, 101 patients (35%) presented RLS, with higher prevalence among females (*p* = 0.032). Compared to RLS-free patients, they showed similar hemodynamics but more severe calf deoxygenation (*p* < 0.001) and lower mobility (*p* = 0.040). Eighty-seven RLS patients (83%) reported the disappearance of symptoms after 39 (36−70) days of exercise. This subgroup, compared to nonresponders, showed higher adherence (*p* < 0.001), hemodynamic (*p* = 0.041), and mobility improvements (*p* = 0.003). RLS symptoms were frequent in PAD but were reduced by a pain-free walking exercise aimed at inducing peripheral aerobic adaptations. The concomitant recovery of sleep and mobility may represent a synergistic action against the cardiovascular risk in PAD.

## 1. Introduction

Restless legs syndrome (RLS), a common sensory-motor disorder with a relatively high prevalence in older people and females, causes unpleasant sensations in patients’ limbs and induces patients to move their legs [1,2,3,4]. Interestingly, RLS symptoms exhibit a distinct circadian pattern, with an increase in both sensory and motor symptoms in the evening and at night [5]. On the one hand, immobility plays an important role, and a worsening of RLS symptoms by nocturnal immobility is closely linked to intrinsic circadian variations [6]. On the other hand, periodic limb movements during sleep show a nocturnal pattern, with most of the movements peaking at the beginning of the night, preceding the evening melatonin rise [7]. The sleep disruptions that occur may be responsible for daytime sleepiness, fatigue [1,8,9], and impaired performance during activities of daily living [10]. Finally, an association between RLS and a high risk of suicide and self-harm, independent of comorbidities and conditions, has been recently reported [11].

To improve the detection of RLS, clinical diagnostic criteria have been established and reviewed [12]. Unfortunately, the effects of the disorder remain underestimated by patients and poorly reported to their doctors [1,2]. In addition to the primary idiopathic form, RLS has been associated with specific conditions (peripheral hypoxia [13], iron deficiency [14], and pregnancy) and several chronic diseases, including neurological diseases, diabetes, cardio-cerebrovascular diseases, or with the presence of more medical comorbidities [5,15,16,17]. In some cases, the symptoms are associated with significant decrements in health-related quality of life and clinical outcomes [2,16,18,19], and the patients may benefit from various pharmacological agents, as well as moderate-intensity exercise [2,18,20,21,22,23].

Because the pathogenesis of RLS may result from an uncoupling between central inhibition and peripheral nerve function, vascular factors such as endothelial dysfunction, changes in the peripheral microvasculature and peripheral hypoxia have been recently studied [3].

Therefore, RLS might represent a poorly estimated problem [24] in patients with peripheral artery disease (PAD), a pathology that is highly prevalent in elderly people and end-stage renal disease patients [25] and characterized by a reduction in the amount of oxygen delivered to the working muscles [26]. Nocturnal extravascular symptoms in PAD patients might be responsible for sleep disruption, which increases the risk for cardiovascular disease and sedentary behavior in these patients [27,28]. Unfortunately, few studies on this topic are available.

The aims of this retrospective study were to assess the frequency of RLS in a PAD population enrolled in a rehabilitation program and to describe the effects of a low-intensity, pain-free exercise program properly developed for PAD patients [29,30] and adapted for frail individuals [31,32,33,34].

We hypothesized that rehabilitation and this type of exercise in particular, due to its favorable muscular and vascular effects [35,36], might improve RLS symptoms in affected patients.

## 2. Materials and Methods

### 2.1. Subjects and Settings

This single-center study retrospectively analyzed a prospectively collected database of 286 PAD patients enrolled between 2015 and 2018 in the Vascular Rehabilitation Program at the University Hospital of Ferrara. Patients were previously screened through a Doppler ultrasound examination at the department of vascular surgery to assess the presence and severity of PAD.

The local ethics committee approved the study, and the tests conducted in this study were a part of the usual care for PAD patients at the University Hospital of Ferrara.

### 2.2. Variables Assessed upon Enrollment

Upon enrollment in the program, information regarding the clinical status and functional impairment of the patients were collected by consulting patients’ medical documents or conducting specific tests and questionnaires.

In particular, anthropometric data (age, sex, and weight), educational levels, cardiovascular risk factors, the use of medications, comorbidities identified through the Charlson Comorbidity Index [37], the location and severity of endovascular lesions, and patients’ reported claudication distance were noted.

In addition, the presence of RLS was determined by the same physician according to the four criteria highlighted by the European Restless Legs Syndrome Study Group (EURLSSG) guidelines [1]. Specifically, if at least one positive answer was obtained, adjunctive questions from the RLS-diagnostic index were posed to diagnose RLS. The patients were then divided into RLS+ and RLS− groups depending on the presence of RLS at baseline.

### 2.3. Outcome Measures

A battery of hemodynamic and functional tests were performed on the same day between 8:30 and 12:30 a.m. in a temperature-controlled environment to determine the hemodynamic severity, the individual degree of muscle deoxygenation occurring while walking, and the functional capacity of each patient.

Hemodynamic assessment: After five minutes of rest, the ankle-brachial index (ABI) was measured according to the standard procedure [38] by a Doppler ultrasound (Stereodop 448. S; Ultrasomed, Milan, Italy) on both limbs. The leg with the lower ABI value was considered the more impaired leg. The vessels were considered “not compressible” for ABI measurements >1.31.

Degree of muscular oxygenation: After a suitable duration of rest, the degree of muscular oxygenation was determined by means of an incremental treadmill test [39] assisted by near-infrared spectroscopy (NIRS) [40]. NIRS sensors were attached to each patient’s medial gastrocnemius muscle. The NIRS system (Oxymon MK III Artinis Medical System, the Netherlands) comprised of 2 channels (2 pulsed light sources and 2 detectors) that emitted infrared light at a frequency of 1 Hz. The intensity of the reflected light provided information about oxygenated hemoglobin (oxy-Hb) concentration.

For the treadmill test, patients walked on level ground at a slow initial speed (1.5 km/h), and small increases to the speed were made (0.1 km/h every 10 m) until the maximal speed (Smax) or a speed that a patient was unable to maintain for any reason (peripheral symptoms, fatigue, or dyspnea) was reached.

During the test, the speed at the onset of claudication symptoms or the pain threshold speed (PTS) was also recorded. At the end of the sessions, NIRS data were analyzed with dedicated software to determine the maximal degree of deoxygenation (or the most negative values reached during the test) and the area under the curve of the oxy-Hb concentration [40].

*Functional measurement*: The 6-min walk test was performed to assess exercise capacity [41]. Patients were asked to walk back and forth on a 20-m corridor for a total duration of 6 min and to cover as much distance as possible in the fixed duration. During the test, the patients were asked to indicate when the onset of claudication symptoms occurred, and they were allowed to stop whenever they needed for leg symptoms or fatigue. The same skilled assessor recorded the total distance walked (6-min walking distance, 6MWD) and the distance at the onset of symptoms (pain-free walking distance) for all patients.

### 2.4. Exercise Program

All patients received the ‘Test in–Train out’ (Ti-To) structured home-based exercise program [29,30], composed of a hospital-based phase and a home-based phase with walking exercises. The hospital-based phase included approximately monthly visits at the hospital for clinical evaluations, including an assessment of RLS presence, ABI and 6MWD measurements, an updated prescription of the home-based program, and an evaluation of the patient’s adherence to the home-based program.

The home-based phase included the execution of exercises at home, preferably indoors. The program was based on two 10-min walking sessions per day (six days per week) of intermittent walking (one minute of walking and one minute of resting while seated) at a prescribed speed; in the beginning, the prescribed speed was slower than the patient’s typical walking speed, and then the prescribed speed increased weekly. The training speed was converted into a walking cadence (steps/minute) that was to be maintained at home with a metronome. The patients were specifically instructed to walk in rhythm with the metronome. Training progression included both speed increases from 60 to 84–100 steps/min according to the severity of claudication and walk–rest ratio increases from 1:1 to 2:1 and 3:1.

Patients were asked to fill out a daily training record indicating completion of the exercises, any related symptoms, and the presence or absence of RLS or cramps each night.

Patients were discharged from the program when they were satisfied with the improvements in pain-free walking distance (PFWD); for example, a patient was discharged when he or she reached a symptom-free walking speed that was normal for his or her age or when claudication symptoms were stable for three consecutive tests. The percentage of completed exercise sessions with respect to the prescribed number of sessions was calculated at the end of the program.

### 2.5. RLS Progression throughout the Program

Patients who experienced RLS at baseline were asked about RLS remission or persistence at each hospital visit. If RLS remission was reported, the number of days since the beginning of the program was calculated.

### 2.6. Statistical Analysis

Continuous data are expressed as the mean ± standard deviation and categorical data are expressed as number and percentage.

RLS+ and RLS− group comparisons were performed by independent *t*-tests (normally distributed data), Mann–Whitney tests (nonnormally distributed data), or chi-squared tests (categorical data), as appropriate. Multiple and logistic regression models with a stepwise selection method were applied to identify factors related to RLS remission after the exercise program. Independent variables considered at baseline were: age, sex, BMI, risk factors and comorbidities (Table 1), disease duration and severity (ABI), Smax, PFWD and 6MWD, and deoxygenation at calves. In addition, for logistic regression an improvement of PFWD and 6MWD arbitrarily set at 25 m and an ABI improvement greater than 0.05 for both limbs were also considered.

Within-group comparisons concerning RLS remission or persistence were assessed by paired *t*-tests (normally distributed data) or Wilcoxon rank tests (nonnormally distributed data), as appropriate. The between-group comparisons of outcome variations obtained at the end of the program were performed with one-way analysis of variance.

A *p*-value of <0.05 was considered statistically significant. All statistical analyses were performed using MedCalc Statistical Software version 19.0.3 (MedCalc Software bvba, Ostend, Belgium). Research data are available at http://dx.doi.org/10.17632/j4dhkwmw3n.1

## 3. Results

A total of 286 patients were evaluated upon enrollment in the rehabilitation program. The characteristics of the population, including anthropometric measures, comorbidities, medications, and PAD-related data, are reported in Table 1.

### 3.1. RLS Prevalence

Upon enrollment, 101 patients (35%) reported having RLS by providing positive answers to the questionnaire. All of the patients presented with nighttime symptoms, and the majority of the patients had intermittent RLS (*n* = 90). Fourteen patients were already treated with a drug suggested by EURLSSG, but they still reported symptoms (Table 1).

At baseline, a higher prevalence of RLS was observed in females than in males (*p* = 0.032), and patients in the RLS+ group showed a lower exercise capacity, measured both on the treadmill and over ground, than the RLS− group did. The comparison between the two groups is reported in Table 2.

In addition, compared to the RLS− group, the RLS+ group showed a significantly higher degree of deoxygenation in both the more impaired limb (−14.1 ± 7.0 arbitrary units (a.u.) for RLS+ versus −7.6 ± 4.6 a.u. for RLS−; *p* < 0.001) and the less impaired limb (−8.2 ± 4.8 a.u. for RLS+ versus −3.8 ± 3.6 a.u. for RLS−; *p* < 0.001).

**Figure 1:**
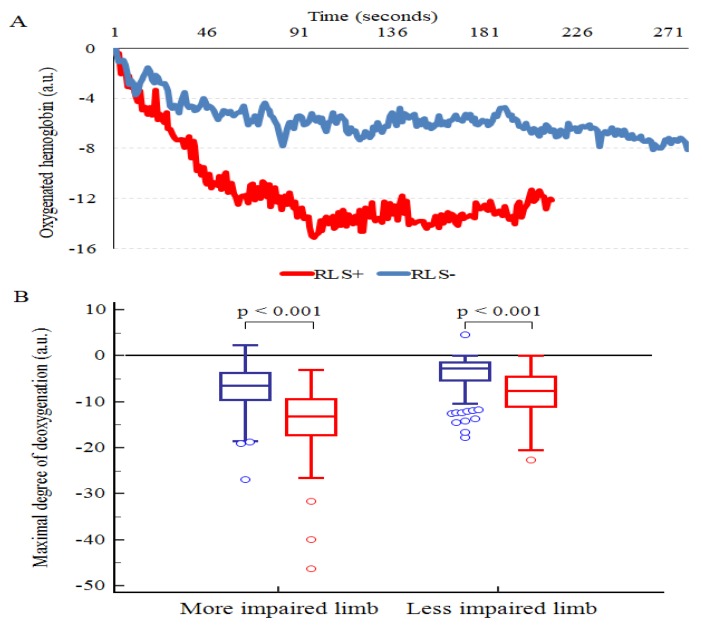
Mean oxyhemoglobin tracks for the more impaired limb (**A**) and maximal degree of deoxygenation of both limbs (**B**) in the two groups at baseline. Legend: red: RLS+; blue: RLS−.

No differences were observed in the area under the curve of the oxygenated hemoglobin in both limbs due to a longer duration of the test observed in the RLS− group than in the RLS+ group. The data are represented in Figure 1.

### 3.2. RLS after the Exercise Program

All patients completed the prescribed exercise program without any adverse events. Within the RLS+ group, 83 patients (82%) reported the disappearance of RLS during the program, while the remaining 18 (18%) did not. RLS remission occurred after a median of 39 days of training (interquartile range: 36–70), corresponding to the time of the first hospital visit after the beginning of the program.

Among the 14 patients who were taking medications for RLS, 13 reported a remission of symptoms after a median of 39 days, while one patient had a persistence of RLS until the end of the program.

Compared with the remaining patients, the subgroup who exhibited RLS remission executed a significantly higher number of exercise sessions, showed greater hemodynamic improvements in the more impaired limb (and significantly larger changes in the PFWD and 6MWD (Table 3).

Within the statistically significant logistic regression model (R^2^ 0.10; *p* = 0.011), the only factor related to the remission of RLS was an increase in the 6MWD greater than 25 m (Odds ratio: 8.00; 95% confidence interval 1.01 to 63.56).

## 4. Discussion

The study revealed a high percentage of PAD patients with claudication suffering from RLS and nocturnal muscular cramps. Among these patients, the presence of nocturnal symptoms was related to low mobility and a high degree of calf deoxygenation while walking during an incremental treadmill test assisted by NIRS. The study also showed that low-intensity pain-free walking exercise was able to reverse the symptoms in a large percentage of the patients enrolled in a structured home-based rehabilitation program [29,30].

The prevalence of nocturnal RLS symptoms has been poorly described in PAD patients. In the general population, according to the analysis derived from three prospective studies, a decreased ABI was not found to be a risk factor for RLS [42]. However, patients with PAD often present comorbidities associated with RLS such as chronic kidney disease (CKD). Among dialysis patients, who often have PAD [2,43], RLS and muscle cramps were reported in 16% to 30% of the patients studied [2,43,44,45], and there was no association between PAD and cramps during dialysis [43,44]. The symptoms that were reported had an age-dependent prevalence [44], and symptoms were reported more frequently in women than in men among dialysis and nondialysis CKD patients [18,45]. RLS was also found to have a variable prevalence (7–24%) in diabetic patients, with poor sleep quality [46,47,48,49], and with nocturnal symptoms; in some cases, RLS was associated with peripheral neuropathy [46].

However, the prevalence of RLS is strongly dependent on the method of assessment: the use of standardized questionnaires only or additional neurological examinations. When a neurological examination or a diagnostic questionnaire which removes RLS mimics were associated to the pool of questions, the prevalence of RLS dropped from 21% to 4.5% among dialysis patients and from 22% to 8% in patients with peripheral neuropathy [18,50].

In the study population of PAD patients, the prevalence of RLS that was determined by the four questions was high (35%). This prevalence, considering the low number of patients under pharmacological treatment, suggests that RLS is poorly estimated or treated in these patients. The prevalence was significantly higher in females than in males, but there were no differences in prevalence with different levels of BMI, with the use of diuretics, with the presence of comorbidities, with different durations of the disease or with different ABI values. In this population, the mobility at baseline measured by a validated test was inversely correlated with the presence of nocturnal symptoms. A relationship between the physical activity level assessed by questionnaires or other health-related quality of life (HRQoL) measures and the perception of physical limitations and pain was also observed in other patients with RLS [8]. Interestingly, among diabetic patients with RLS, a higher physical activity level was associated with better HRQoL measures, less pain, and physical limitations. [8].

Our study supports the previous findings of Salminen et al. [13] who noninvasively measured the peripheral oxygen partial pressures on the skin of the legs observing an association between peripheral hypoxia and appearance of RLS symptoms. Moreover, the present study adds an additional element of discussion, because of the beneficial use of the NIRS technique, which allowed a dynamic noninvasive study of muscle metabolism inside the tissue and not only on the skin and during a standardized walking task and not only at rest [40]. This technique revealed that patients with a high degree of muscle deoxygenation in both limbs while walking rather than those that had more severe PAD were suffering more from RLS. This observation is interesting for different reasons. The first reason is that the presence of a new objective parameter correlated with the symptoms. According to some authors, the lack of objective diagnostic findings (e.g., neurological tests) may be attributed to the psychogenic origin of these symptoms [27]. Second, NIRS—unlike a static measure of hemodynamic delivery such as ABI—explores the dynamic imbalance between oxygen request and both delivery and utilization in the muscles.

This analysis more closely describes the aerobic inefficiency of muscles (collaterals, capillaries, microcirculation, and mitochondrial activity) [51] that is potentially associated with chronic peripheral fatigue. Among the multiple mechanisms that contribute to the pathogenesis of RLS, metabolic factors (hypoxia and muscle fatigue) may affect peripheral nerve function and alter both central and especially peripheral excitability in individuals with RLS [19]. Interestingly, performing physical activity just before going to bed has been linked to an increase in RLS symptoms [8,52]. Other factors involved in oxygen delivery have also been associated with RLS, such as poor vascular endothelial function [3], altered brachial arterial pulse wave velocity in patients with acute ischemic stroke [16] or greater peripheral vascular resistance [53]. Conversely, the administration of drugs with vasodilating effects such as cilostazol partially improved the clinical symptoms of RLS [25]. Finally, low iron and ferritin concentrations, conditions potentially associated with anemia and reduced oxygen transport, have also been considered factors that trigger RLS [14].

Finally, the study supports the role of exercise in managing these nocturnal symptoms. In dialysis patients, in addition to medications (dopamine agonists, benzodiazepines, antiepileptics, iron, and vitamins), exercise, particularly aerobic exercise, reduced RLS symptoms in CKD patients [54,55,56]. Improvements in insomnia and RLS symptoms and HRQoL were also reported after exercise in hemodialysis patients [2,21,22,23,57,58]. Aerobic exercise could reduce RLS symptoms [9,12,23,55,59] by evoking favorable adaptations in aging skeletal muscles [60], increasing endorphin production [61,62,63], improving thermoregulation, which favors sleep [2,57], or inducing vasodilation with improvements in blood supply [2,64].

In our study, the RLS symptoms were significantly and rapidly reduced after many repetitions of the same type of low-intensity exercise, which previously induced peripheral hemodynamic and muscle aerobic adaptations, as highlighted by the NIRS technique, in PAD and in dialysis patients [35,36]. This intervention potentially has a summation effect on pharmacological therapy, considering that almost all patients that were taking the prescribed RLS drugs showed partial benefits from the drugs reported remission of their symptoms after a few weeks of training.

The reduction in symptoms observed with rehabilitation represents the removal of an additional cardiovascular risk factor in a population at high risk for cardiovascular disease. An overactivity of the nocturnal HPA system, characterized by significantly enhanced nocturnal cortisol excretion, has been shown in RLS subjects. Such hyperactivity has been considered a possible mechanism contributing to the increased prevalence of cardiovascular disease in RLS patients than in control subjects [65]. In fact, patients with RLS have been shown to show higher nocturnal and sleep-time systolic blood pressure compared to control subjects with no sleep disorders [66]. Moreover, hypertension and RLS symptoms seem to be linked since patients with nondipping blood pressure patterns (NDBPP) had higher RLS symptom scores and more severe RLS symptoms than patients who had RLS but not NDBPP [67]. Sleep fragmentation and deprivation altered circadian rhythms with proinflammatory and proatherosclerotic effects [27,68]. As a consequence, mood disturbance and loss of performance due to sleep disruption may negatively affect the willingness and capacity to exercise during rehabilitation [2].

In addition to the strengths of our study, which include the large sample size and novel use of NIRS technology for improving scientific knowledge, a number of limitations remain in this retrospective study, without a control group. RLS was assessed by a few questions, during a face-to-face interview, without confirmation by diagnostic tests or the involvement of sleep specialists. This limitation is relevant because nocturnal leg cramps are common in older people and are associated with many common diseases and medications [20]. Moreover, in addition to the diagnostic procedure, the presence of chronic pain or population differences may have affected the results of the study [50]. Finally, nor the family history of RLS or the presence of periodic limb movements, nor the RLS severity were evaluated. RLS was also not correlated to the degree of PAD, and secondary RLS related to CKD and iron deficiency anemia were not assessed.

## 5. Conclusions

The prevalence of RLS as assessed by a standardized number of questions was high in a PAD population with claudication. The symptoms were not related to disease severity measured at rest but were related to the degree of metabolic fatigue in muscles assessed during treadmill walking. The remission of nocturnal symptoms in several patients by the execution of low-intensity exercise supports the effectiveness of this intervention, at least in the presence of a muscle-altered metabolic status. However, this preliminary observation needs to be confirmed in prospective studies with neurological evaluations.

## Figures and Tables

**Table 1:** Characteristics of the population included in the study.

	Whole Population (*n* = 286)
Age, years	71 ± 9
Males, *n* (%)	219 (77)
BMI (kg·m^−2^)	28 ± 5
Risk factors, *n* (*%*)	
Smoking	239 (84)
Hypertension	246 (86)
Dyslipidemia	215 (75)
Diabetes	138 (48)
Familiarity for CVD	26 (9)
*Comorbidities*, *n* (*%*)	
Coronary artery disease	111 (39)
Lung disease	49 (17)
Osteoarticular disorders	71 (25)
Chronic kidney disease	51 (18)
Cerebrovascular disease	29 (10)
Lower limbs revascularizations	78 (27)
Charlson Index	6.3 ± 2.0
*Therapy*, *n* (*%*)	
Anticoagulants	32 (11)
Antiplatelet	261 (91)
Anti-hypertensive	246 (86)
Diuretics	90 (31)
Statins	212 (74)
Hypoglycemic agents and/or insulin	138 (48)
RLS-treatment drugs	18 (6)
Levodopa	1 (0.00)
Ropinirole	0 (0)
Pramipexole	2 (0.01)
Rotigotine	0 (0)
Pregabalin	6 (2)
Clonazepam	3 (0.01)
Gabapentin	2 (0.01)
*Peripheral artery disease*	
Duration, years	4 ± 7
ABI more diseased limb	0.68 ± 0.21
ABI less diseases limb	0.84 ± 0.19
Speed at symptoms (km·h^−1^)	2.7 ± 0.9
Maximal speed (km·h^−1^)	3.3 ± 1.0
Pain-free walking distance (m)	142 ± 83
6-min walking distance (m)	313 ± 81

Abbreviations: ABI, ankle-brachial index.

**Table 2:** Characteristics of the population included in the study.

	RLS+ (*n* = 101)	RLS− (*n* = 185)	*p*
Age, years	71 ± 9	72 ± 9	0.51
Males, *n* (%)	70 (69)	149 (81)	0.032
BMI (kg·m^−2^)	28 ± 5	27 ± 5	0.76
*Risk factors*, *n* (*%*)			
Smoking	82 (81)	157 (85)	0.42
Hypertension	74 (73)	141 (76)	0.58
Dyslipidemia	74 (73)	141 (76)	0.58
Diabetes	52 (52)	86 (47)	0.42
Familiarity for CVD	8 (8)	18 (10)	0.61
*Comorbidities*, *n* (*%*)			
Coronary artery disease	36 (36)	75 (41)	0.42
Lung disease	17 (17)	32 (17)	0.88
Osteoarticular disorders	23 (23)	48 (26)	0.55
Chronic kidney disease	24 (24)	27 (15)	0.06
Cerebrovascular disease	10 (10)	19 (10)	0.79
Lower limbs revascularizations	32 (32)	46 (25)	0.21
Charlson Index	6.4 ± 2.0	6.2 ± 2.0	0.41
*Therapy*, *n* (*%*)			
Anticoagulants	10 (10)	22 (11)	0.67
Antiplatelet	90 (89)	171 (92)	0.55
Anti-hypertensive	74 (73)	141 (76)	0.58
Diuretics	38 (38)	52 (28)	0.10
Statins	74 (73)	141 (76)	0.58
Hypoglycemic agents and/or insulin	52 (52)	86 (47)	0.42
RLS-treatment drugs	14 (14)	4 (2)	<0.001
*Peripheral artery disease*			
Duration, years	4 ± 4	4 ± 7	0.81
ABI more diseased limb	0.69 ± 0.22	0.68 ± 0.20	0.83
ABI less diseases limb	0.83 ± 0.19	0.84 ± 0.20	0.77
Speed at symptoms (km·h^−1^)	2.5 ± 0.8	2.8 ± 1.0	0.018
Maximal speed (km·h^−1^)	3.1 ± 1.0	3.4 ± 1.0	0.032
Pain-free walking distance (m)	132 ± 90	148 ± 70	0.12
6-min walking distance (m)	300 ± 75	320 ± 84	0.044

Abbreviations: ABI, ankle-brachial index.

**Table 3:** Outcomes of the exercise program within restless leg syndrome (RLS)+ group of patients who either did or did not exhibit RLS remission.

	RLS Remission (*n* = 83)	RLS Persistence (*n* = 18)	*p*
Program duration (days)	288 ± 121	304 ± 117	0.23
Exercise sessions executed (%)	94	71	<0.001
∆ ABI more diseased limb	0.05 ± 0.11	0.00 ± 0.12	0.041
∆ ABI less diseases limb	0.03 ± 0.14	0.02 ± 0.12	0.43
∆ Pain-free walking distance (m)	82 ± 79	40 ± 88	0.049
∆ 6-min walking distance (m)	20 ± 43	−11 ± 32	0.003

Abbreviations: ABI, ankle-brachial index.

## References

[1] Garcia-Borreguero D., Stillman P., Benes H., Buschmann H., Chaudhuri K.R., Gonzalez Rodríguez V.M., Högl B., Kohnen R., Monti G.C., Stiasny-Kolster K. (2011). Algorithms for the diagnosis and treatment of restless legs syndrome in primary care. BMC Neurol..

[2] Anand S., Johansen K.L., Grimes B., Kaysen G.A., Dalrymple L.S., Kutner N.G., Chertow G.M. (2013). Physical activity and self-reported symptoms of insomnia; restless legs syndrome; and depression: The comprehensive dialysis study. Hemodial. Int..

[3] Koh S.Y., Kim M.S., Lee S.M., Hong J.M., Yoon J.H. (2015). Impaired vascular endothelial function in patients with restless legs syndrome: A new aspect of the vascular pathophysiology. J. Neurol. Sci..

[4] Guo S., Huang J., Jiang H., Han C., Li J., Xu X., Zhang G., Lin Z., Xiong N., Wang T. (2017). Restless Legs Syndrome: From Pathophysiology to Clinical Diagnosis and Management. Front. Aging Neurosci..

[5] Garcia-Borreguero D., Larrosa O., de la Llave Y. (2002). Circadian aspects in the pathophysiology of the restless legs syndrome. Sleep Med..

[6] Michaud M., Dumont M., Paquet J., Desautels A., Fantini M.L., Montplaisir J. (2005). Circadian variation of the effects of immobility on symptoms of restless legs syndrome. Sleep.

[7] Duffy J.F., Lowe A.S., Silva E.J., Winkelman J.W. (2011). Periodic limb movements in sleep exhibit a circadian rhythm that is maximal in the late evening/early night. Sleep Med..

[8] Daniele T.M., de Bruin V.M., e Forte A.C., de Oliveira D.S., Pompeu C.M., de Bruin P.F. (2013). The relationship between physical activity; restless legs syndrome; and health-related quality of life in type 2 diabetes. Endocrine.

[9] Cuellar N.G., Ratcliffe S.J. (2008). A comparison of glycemic control; sleep; fatigue; and depression in type 2 diabetes with and without restless legs syndrome. J. Clin. Sleep Med..

[10] Allen R.P., Walters A.S., Montplaisir J., Hening W., Myers A., Bell T.J., Ferini-Strambi L. (2005). Restless legs syndrome prevalence and impact: REST general population study. Arch. Intern. Med..

[11] Zhuang S., Na M., Winkelman J.W., Ba D., Liu C.F., Liu G., Gao X. (2019). Association of Restless Legs Syndrome With Risk of Suicide and Self-harm. JAMA Netw. Open.

[12] Allen R.P., Picchietti D., Hening W.A., Trenkwalder C., Walters A.S., Montplaisi J., Restless Legs Syndrome Diagnosis and Epidemiology workshop at the National Institutes of Health, International Restless Legs Syndrome Study Group (2003). Restless legs syndrome: Diagnostic criteria; special considerations; and epidemiology. A report from the restless legs syndrome diagnosis and epidemiology workshop at the National Institutes of Health. Sleep Med..

[13] Salminen A.V., Rimpilä V., Polo O. (2014). Peripheral hypoxia in restless legs syndrome (Willis-Ekbom disease). Neurology.

[14] Jiménez-Jiménez F.J., Alonso-Navarro H., García-Martín E., Agúndez J.A.G. (2019). Neurochemical features of idiopathic restless legs syndrome. Sleep Med. Rev..

[15] Trenkwalder C., Allen R., Högl B., Paulus W., Winkelmann J. (2016). Restless legs syndrome associated with major diseases: A systematic review and new concept. Neurology.

[16] Han S.H., Park K.Y., Kim J.M., Youn Y.C., Shin H.W. (2019). Restless legs syndrome is associated with arterial stiffness and clinical outcome in stroke patients. Sleep Med..

[17] Szentkirályi A., Völzke H., Hoffmann W., Trenkwalder C., Berger K. (2014). Multimorbidity and the risk of restless legs syndrome in 2 prospective cohort studies. Neurology.

[18] Calviño J., Cigarrán S., Lopez L.M., Martinez A., Sobrido M.J. (2015). Restless legs syndrome in non-dialysis renal patients: Is it really that common?. J. Clin. Sleep Med..

[19] Lanza G., Bachmann C.G., Ghorayeb I., Wang Y., Ferri R., Paulus W. (2017). Central and peripheral nervous system excitability in restless legs syndrome. Sleep Med..

[20] Butler J.V., Mulkerrin E.C., O’Keeffe S.T. (2002). Nocturnal leg cramps in older people. Postgrad. Med. J..

[21] King A.C., Pruitt L.A., Woo S., Castro C.M., Ahn D.K., Vitiello M.V., Woodward S.H., Bliwise D.L. (2008). Effects of moderate-intensity exercise on polysomnographic and subjective sleep quality in older adults with mild to moderate sleep complaints. J. Gerontol. A Biol. Sci. Med. Sci..

[22] King A.C., Oman R.F., Brassington G.S., Bliwise D.L., Haskell W.L. (1997). Moderate-intensity exercise and self-rated quality of sleep in older adults. A randomized controlled trial. JAMA.

[23] Aukerman M.M., Aukerman D., Bayard M., Tudiver F., Thorp L., Bailey B. (2006). Exercise and restless legs syndrome: A randomized controlled trial. J. Am. Board Fam. Med..

[24] Abdulla A.J., Jones P.W., Pearce V.R. (1999). Leg cramps in the elderly: Prevalence; drug and disease associations. Int. J. Clin. Pract..

[25] Shiohira S., Yoshida T., Sugiura H., Yoshida S., Mitobe M., Shimada K., Ohba T., Tsuchiya K., Kabaya T., Nitta K. (2011). Effect of the antiplatelet agent cilostazol on endovascular inflammatory biochemical parameters and the clinical symptoms of peripheral artery disease and restless legs syndrome in hemodialysis patients. Clin. Exp. Nephrol..

[26] Gerhard-Herman M.D., Gornik H.L., Barrett C., Barshes N.R., Corriere M.A., Drachman D.E., Fleisher L.A., Fowkes F.G.R., Hamburg N.M., Writing Committee Members (2017). ACC/AHA Task Force Members. 2016 AHA/ACC Guideline on the Management of Patients with Lower Extremity Peripheral Artery Disease: Executive Summary. Vasc. Med..

[27] Silvani A. (2019). Sleep disorders; nocturnal blood pressure; and cardiovascular risk: A translational perspective. Auton. Neurosci..

[28] Zanigni S., Calandra-Buonaura G., Giannini G., Tonon C., Cortelli P., Provini F. (2015). The association between restless legs syndrome; cardiovascular and metabolic diseases: Hypotheses and evidence from the literature. Arch. Ital. Biol..

[29] Manfredini F., Malagoni A.M., Mascoli F., Mandini S., Taddia M.C., Basaglia N., Manfredini R., Conconi F., Zamboni P. (2008). Training rather than walking: The test in -train out program for home-based rehabilitation in peripheral arteriopathy. Circ. J..

[30] Malagoni A.M., Vagnoni E., Felisatti M., Mandini S., Heidari M., Mascoli F., Basaglia N., Manfredini R., Zamboni P., Manfredini F. (2011). Evaluation of patient compliance; quality of life impact and cost-effectiveness of a “test in-train out” exercise-based rehabilitation program for patients with intermittent claudication. Circ. J..

[31] Malagoni A.M., Cavazza S., Ferraresi G., Grassi G., Felisatti M., Lamberti N., Basaglia N., Manfredini F. (2016). Effects of a “test in-train out” walking program versus supervised standard rehabilitation in chronic stroke patients: A feasibility and pilot randomized study. Eur. J. Phys. Rehabil. Med..

[32] Lamberti N., Straudi S., Malagoni A.M., Argirò M., Felisatti M., Nardini E., Zambon C., Basaglia N., Manfredini F. (2017). Effects of low-intensity endurance and resistance training on mobility in chronic stroke survivors: A pilot randomized controlled study. Eur. J. Phys. Rehabil. Med..

[33] Malagoni A.M., Catizone L., Mandini S., Soffritti S., Manfredini R., Boari B., Russo G., Basaglia N., Zamboni P., Manfredini F. (2008). Acute and long-term effects of an exercise program for dialysis patients prescribed in hospital and performed at home. J. Nephrol..

[34] Manfredini F., Mallamaci F., D’Arrigo G., Baggetta R., Bolignano D., Torino C., Lamberti N., Bertoli S., Ciurlino D., Rocca-Rey L. (2017). Exercise in Patients on Dialysis: A Multicenter; Randomized Clinical Trial. J. Am. Soc. Nephrol..

[35] Manfredini F., Malagoni A.M., Mandini S., Felisatti M., Mascoli F., Basaglia N., Manfredini R., Mikhailidis D.P., Zamboni P. (2012). Near-infrared spectroscopy assessment following exercise training in patients with intermittent claudication and in untrained healthy participants. Vasc. Endovascular. Surg..

[36] Manfredini F., Lamberti N., Malagoni A.M., Felisatti M., Zuccalà A., Torino C., Tripepi G., Catizone L., Mallamaci F., Zoccali C. (2015). The role of deconditioning in the end-stage renal disease myopathy: Physical exercise improves altered resting muscle oxygen consumption. Am. J. Nephrol..

[37] Charlson M., Szatrowski T.P., Peterson J., Gold J. (1994). Validation of a combined comorbidity index. J. Clin. Epidemiol..

[38] Aboyans V., Ricco J.B., Bartelink M.E.L., Björck M., Brodmann M., Cohnert T., Collet J.P., Czerny M., De Carlo M., Debus S. (2018). 2017 ESC Guidelines on the Diagnosis and Treatment of Peripheral Arterial Diseases; in collaboration with the European Society for Vascular Surgery (ESVS): Document covering atherosclerotic disease of extracranial carotid and vertebral; mesenteric; renal; upper and lower extremity arteriesEndorsed by: The European Stroke Organization (ESO)The Task Force for the Diagnosis and Treatment of Peripheral Arterial Diseases of the European Society of Cardiology (ESC) and of the European Society for Vascular Surgery (ESVS). Eur. Heart J..

[39] Manfredini F., Conconi F., Malagoni A.M., Manfredini R., Mascoli F., Liboni A., Zamboni P. (2004). Speed rather than distance: A novel graded treadmill test to assess claudication. Eur. J. Vasc. Endovasc. Surg..

[40] Manfredini F., Malagoni A.M., Felisatti M., Mandini S., Mascoli F., Manfredini R., Basaglia N., Zamboni P. (2009). A dynamic objective evaluation of peripheral arterial disease by near-infrared spectroscopy. Eur. J. Vasc. Endovasc. Surg..

[41] Montgomery P.S., Gardner A.W. (1998). The clinical utility of a six-minute walk test in peripheral arterial occlusive disease patients. J. Am. Geriatr. Soc..

[42] Szentkirályi A., Völzke H., Hoffmann W., Dörr M., Hense H.W., Berger K. (2017). Ankle-brachial index and peripheral artery disease are not related to restless legs syndrome. Sleep Med..

[43] Ghimire M., Sharma S.K., Chimoriya R., Das G.C. (2014). Intradialytic Muscle Cramp and its Association with Peripheral Arterial Disease in End Stage Renal Disease Patients on Hemodialysis. JNMA J. Nepal. Med. Assoc..

[44] Brass E.P., Adler S., Sietsema K.E., Amato A., Esler A., Hiatt W.R. (2002). Peripheral arterial disease is not associated with an increased prevalence of intradialytic cramps in patients on maintenance hemodialysis. Am. J. Nephrol..

[45] Stefanidis I., Vainas A., Dardiotis E., Giannaki C.D., Gourli P., Papadopoulou D., Vakianis P., Patsidis E., Eleftheriadis T., Liakopoulos V. (2013). Restless legs syndrome in hemodialysis patients: An epidemiologic survey in Greece. Sleep Med..

[46] Lopes L.A., Lins Cde M., Adeodato V.G., Quental D.P., de Bruin P.F., Montenegro R.M., de Bruin V.M. (2005). Restless legs syndrome and quality of sleep in type 2 diabetes. Diabetes Care.

[47] O’Hare J.A., Abuaisha F., Geoghegan M. (1994). Prevalence and forms of neuropathic morbidity in 800 diabetics. Ir. J. Med. Sci..

[48] Merlino G., Fratticci L., Valente M., Del Giudice A., Noacco C., Dolso P., Cancelli I., Scalise A., Gigli G.L. (2007). Association of restless legs syndrome in type 2 diabetes: A case-control study. Sleep.

[49] Skomro R.P., Ludwig S., Salamon E., Kryger M.H. (2001). Sleep complaints and restless legs syndrome in adult type 2 diabetics. Sleep Med..

[50] Cho Y.W., Na G.Y., Lim J.G., Kim S.H., Kim H.S., Earley C.J., Allen R.P. (2013). Prevalence and clinical characteristics of restless legs syndrome in diabetic peripheral neuropathy: Comparison with chronic osteoarthritis. Sleep Med..

[51] Manfredini F., Lamberti N., Rossi T., Mascoli F., Basaglia N., Zamboni P. (2017). A Toe Flexion NIRS assisted Test for Rapid Assessment of Foot Perfusion in Peripheral Arterial Disease: Feasibility, Validity, and Diagnostic Accuracy. Eur. J. Vasc. Endovasc. Surg..

[52] Ohayon M.M., Roth T. (2002). Prevalence of restless legs syndrome and periodic limb movement disorder in the general population. J. Psychosom. Res..

[53] Bertisch S.M., Muresan C., Schoerning L., Winkelman J.W., Taylor J.A. (2016). Impact of Restless Legs Syndrome on Cardiovascular Autonomic Control. Sleep.

[54] Giannaki C.D., Sakkas G.K., Karatzaferi C., Maridaki M.D., Koutedakis Y., Hadjigeorgiou G.M., Stefanidis I. (2015). Combination of Exercise Training and Dopamine Agonists in Patients with RLS on Dialysis: A Randomized; Double-Blind Placebo-Controlled Study. ASAIO J..

[55] Mortazavi M., Vahdatpour B., Ghasempour A., Taheri D., Shahidi S., Moeinzadeh F., Dolatkhah B., Dolatkhah S. (2013). Aerobic exercise improves signs of restless leg syndrome in end stage renal disease patients suffering chronic hemodialysis. Sci. World J..

[56] Gopaluni S., Sherif M., Ahmadouk N.A. (2016). Interventions for chronic kidney disease-associated restless legs syndrome. Cochrane Database Syst. Rev..

[57] Driver H.S., Taylor S.R. (2000). Exercise and sleep. Sleep Med. Rev..

[58] Phillips B., Young T., Finn L., Asher K., Hening W.A., Purvis C. (2000). Epidemiology of restless legs symptoms in adults. Arch. Intern. Med..

[59] Giannaki C.D., Sakkas G.K., Karatzaferi C., Hadjigeorgiou G.M., Lavdas E., Kyriakides T., Koutedakis Y., Stefanidis I. (2013). Effect of exercise training and dopamine agonists in patients with uremic restless legs syndrome: A six-month randomized, partially double-blind, placebo-controlled comparative study. BMC Nephrol..

[60] Forbes S.C., Little J.P., Candow D.G. (2012). Exercise and nutritional interventions for improving aging muscle health. Endocrine.

[61] Von Spiczak S., Whone A.L., Hammers A., Asselin M.C., Turkheimer F., Tings T., Happe S., Paulus W., Trenkwalder C., Brooks D.J. (2005). The role of opioids in restless legs syndrome: An [11C] diprenorphine PET study. Brain.

[62] Walters A.S., Winkelmann J., Trenkwalder C., Fry J.M., Kataria V., Wagner M., Sharma R., Hening W., Li L. (2001). Long-term follow-up on restless legs syndrome patients treated with opioids. Mov. Disord..

[63] Walters A.S., Ondo W.G., Zhu W., Le W. (2009). Does the endogenous opiate system play a role in the Restless Legs Syndrome? A pilot post-mortem study. J. Neurol. Sci..

[64] Mitchell U.H. (2011). Nondrug-related aspect of treating Ekbom disease; formerly known as restless legs syndrome. Neuropsychiatr. Dis. Treat..

[65] Schilling C., Schredl M., Strobl P., Deuschle M. (2010). Restless legs syndrome: Evidence for nocturnal hypothalamic-pituitary-adrenal system activation. Mov. Disord..

[66] Sieminski M., Partinen M. (2016). Nocturnal systolic blood pressure is increased in restless legs syndrome. Sleep Breath..

[67] Ulu S.M., Ahsen A., Akcı Ö., Yaman F., Demir K., Yaman G., Yüksel Ş., Acartürk G. (2015). The relationship between dipping-non-dipping arterial blood pressure pattern and frequency of restless leg syndrome with related factors. Anatol. J. Cardiol..

[68] Gottlieb D.J., Somers V.K., Punjabi N.M., Winkelman J.W. (2017). Restless legs syndrome and cardiovascular disease: A research roadmap. Sleep Med..

